# The Impact of Learning Technologies on the Learning Environment of Medical Students in Africa: A Review of the Current Literature

**DOI:** 10.7759/cureus.97467

**Published:** 2025-11-21

**Authors:** Daniel Thomas, Soo Young Baik, Olivia Beazer, Faraz Sharif, Muhammad Raheel

**Affiliations:** 1 Urology, Royal Hallamshire Hospital, Sheffield, GBR; 2 General and Colorectal Surgery, Mid Yorkshire Teaching NHS Trust, Wakefield, GBR; 3 Medicine, Harrogate and District NHS Foundation Trust, Harrogate, GBR; 4 Internal Medicine, Mid Yorkshire Teaching NHS Trust, Wakefield, GBR; 5 General Surgery, Mid Yorkshire Teaching NHS Trust, Wakefield, GBR

**Keywords:** africa (including madagascar), digital learning platforms, e-learning, medical school students, supportive learning environment, technology in learning, undergraduate-medical students

## Abstract

Medical education continues to evolve rapidly, reshaping how future health professionals are trained and supported. This evolution reflects broader global trends toward learner-centred, competency-based, and technology-enhanced education. Broadly defined, learning technologies are digital tools, platforms, and systems that support teaching, learning, and assessment. While learning environments refer to the physical, social, psychological, and digital spaces where education occurs. This study systematically reviewed the impact of learning technologies within the learning environment of medical students in Africa.

Embase, Ovid, and Emcare databases were queried for relevant research articles regarding learning technologies for medical students in undergraduate medical schools in Africa. Eligible studies involved the implementation of a form of digital technology for medical students. Two independent reviewers selected appropriate literature according to specified inclusion and exclusion criteria.

A total of 14 articles were identified for this review. The included studies in the review were conducted across seven African countries. The majority of studies originated from South Africa (n = 4), followed by Nigeria (n = 3) and Egypt (n = 3). Other regions represented were Uganda (n = 1), Kenya (n = 1), Zambia (n = 1), and Somaliland (n = 1). A wide variety of digital learning technologies were employed across the included studies. The most frequently used were live conferencing platforms such as Zoom (Zoom Video Communications, San Jose, CA), WizIQ (WizIQ Pvt. Ltd., Bangalore, India), and YouTube (Google LLC, Mountain View, CA). Multiple studies concluded that blended approaches were preferred and superior to either fully online or traditional teaching, bridging gaps between theory and practice while enhancing engagement.

Learning technologies hold enormous potential to transform medical education in Africa, enhancing access, equity, and quality of training, whilst also improving the learning environment. Yet this potential remains constrained by systemic barriers, from infrastructure limitations to pedagogical gaps.

## Introduction and background

Medical education continues to evolve rapidly, reshaping how future health professionals are trained and supported. This evolution reflects broader global trends toward learner-centred, competency-based, and technology-enhanced education [1]. In this context, learning technologies, defined as digital tools, platforms, and systems that support teaching, learning, and assessment, have become central to modern medical training [2]. They encompass a broad range of innovations, from simple lecture capture systems and learning management platforms to advanced virtual reality simulations and telemedicine interfaces.

Learning environments, meanwhile, refer to the physical, social, psychological, and digital spaces where education occurs [3]. These environments shape students’ engagement, motivation, and academic outcomes, influencing how effectively they acquire and apply knowledge. Increasingly, educational institutions aim to create blended learning environments-integrating face-to-face and online instruction - to promote active, flexible, and inclusive learning [4]. This review specifically evaluates the impact of learning technologies on the educational environment of medical students in Africa. 

Globally, learning technologies have redefined how medicine is taught, enhancing access, communication, and fidelity in clinical training. Yet the feasibility, sustainability, and scalability of such innovations differ widely between regions. In Africa, where many medical schools historically relied on teacher-centred and lecture-based instruction, digital transformation has been uneven and often constrained by infrastructural and resource limitations [5].

The COVID-19 pandemic accelerated this shift dramatically. When lockdowns forced the rapid move to online teaching, institutions across Africa adopted digital platforms out of necessity rather than long-term strategic planning [6]. This sudden transition exposed long-standing inequities in connectivity, infrastructure, and digital literacy. Many educators and students lacked reliable access to the internet, electricity, and devices, while limited institutional capacity hindered the creation of sustainable e-learning systems [7]. Surveys and case studies across African countries reveal a mix of enthusiasm and frustration: while learners welcomed the flexibility of digital platforms, they often reported dissatisfaction with technical support, limited resources, and insufficient digital skills [8].

Despite these challenges, digital adoption in medical education across Africa continues to grow. Numerous initiatives-including e-learning programmes, mobile-based applications, telemedicine training, and international partnerships-demonstrate the potential of technology to enhance access and collaboration. For instance, postgraduate students in Ethiopia reported limited satisfaction with e-learning resources, while in Nigeria, nearly 80% of health professionals lacked personal computers or adequate digital literacy [9]. Such findings underscore both the promise and the persistence of barriers to equitable technology integration.

Historically, medical education in Africa has been characterised by didactic, teacher-centred methods, constrained clinical exposure, and limited access to educational resources [10]. While this model has supported the training of large cohorts in resource-limited settings, it often restricts active learning, critical thinking, and hands-on experience. The increasing burden of disease, healthcare workforce shortages, and the demand for competency-based education have collectively driven reforms aimed at modernising curricula and teaching strategies [11]. The COVID-19 pandemic served as an accelerant for these changes, transforming digital integration from an aspiration into an urgent necessity [12].

The benefits of learning technologies in medical education are well-documented. They expand access to knowledge by overcoming geographical and financial barriers. Digital libraries and open-access databases, such as HINARI, have democratised access to biomedical literature for students and educators in low- and middle-income countries (LMICs) [13]. Learning technologies also enhance flexibility, allowing students to engage with materials asynchronously, adapt to their own pace, and participate in remote or rural locations [14]. Simulation-based learning tools, ranging from low-cost mannequins to sophisticated virtual and augmented reality platforms, offer safe, reproducible opportunities for skills acquisition, particularly in settings with limited patient exposure [15]. Moreover, digital technologies promote collaboration and global engagement, connecting African medical students with international peers and experts through webinars, massive open online courses (MOOCs), and virtual research networks [16].

At the same time, the learning environment, which includes not only digital access but also social, psychological, and institutional dimensions, remains critical to successful educational outcomes [17]. Student-centred, inclusive, and supportive environments are associated with better academic performance, professional identity formation, and well-being [18]. Digital transformation in education, therefore, must consider how technology reshapes these broader contextual factors, influencing motivation, interaction, and equity among learners.

In recent years, increasing attention has been paid to aligning medical education in Africa with global standards through pedagogical innovations such as problem-based learning (PBL), team-based learning (TBL), and blended learning models [19]. These approaches integrate technology with traditional instruction to encourage active participation, self-directed learning, and collaborative problem-solving. However, the extent to which such technologies improve the learning environment, beyond mere content delivery, remains underexplored in the African context.

While international literature has examined the educational impact of learning technologies extensively, there is a lack of a comprehensive synthesis focusing specifically on African medical education. Existing studies vary widely in methodology, technological focus, and institutional context, limiting the ability to draw generalisable conclusions [20]. Moreover, many evaluations emphasise implementation challenges rather than outcomes related to learning environment quality or student experience.

Therefore, this systematic review aims to examine the current literature on the impact of learning technologies on the learning environment of medical students in Africa. It seeks to identify the types of technologies implemented, their perceived benefits and limitations, and how they influence the physical, social, psychological, and digital dimensions of the learning environment. By synthesising current evidence, this review addresses a critical gap in understanding how technology integration affects medical education in resource-limited contexts and provides insight for policymakers, educators, and researchers striving to strengthen medical training across the continent.

## Review

Methods

Search Strategy

**Figure 1:**
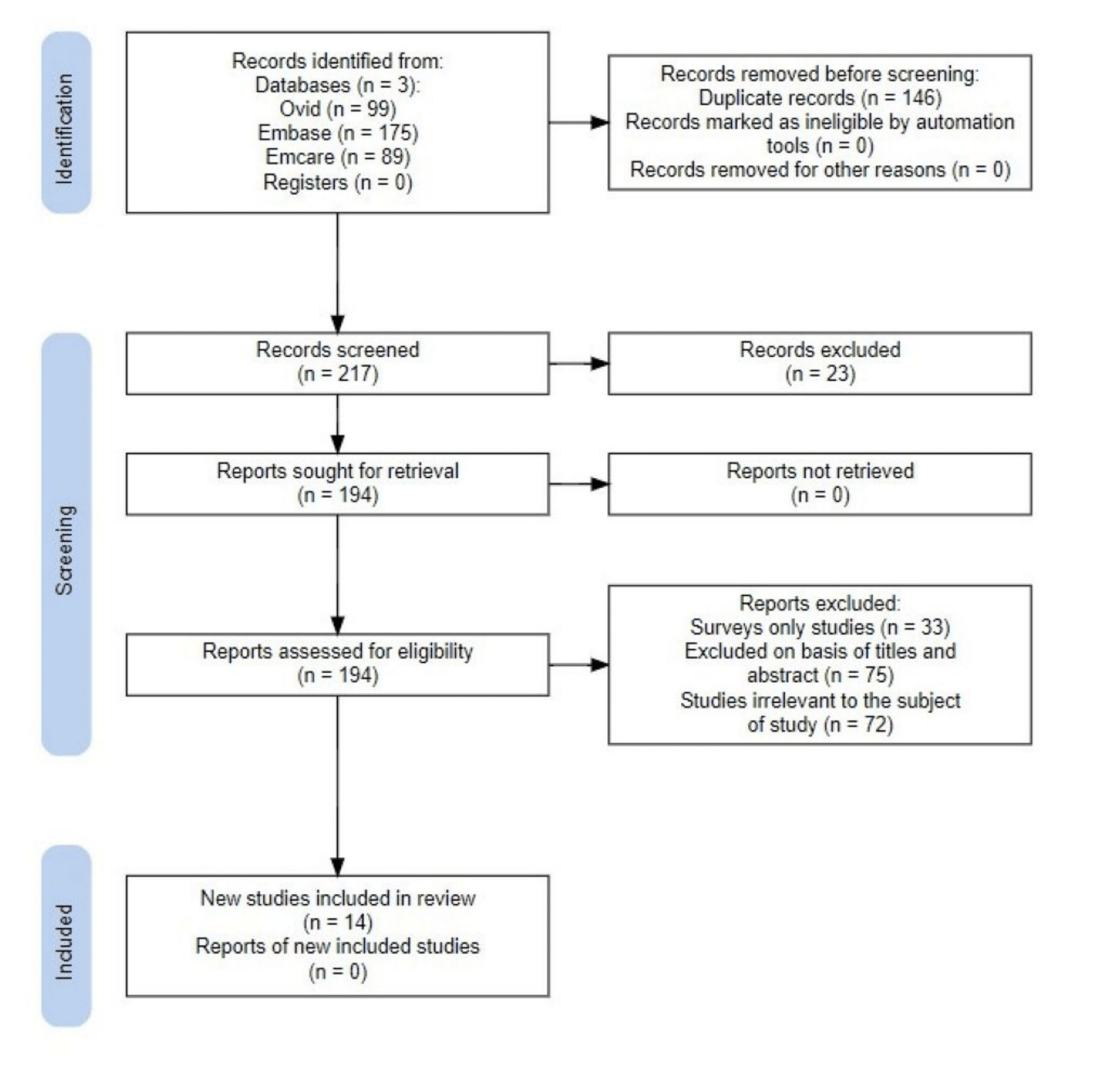
PRISMA diagram showing identification of studies PRISMA: Preferred Reporting Items for Systematic Reviews and Meta-Analyses

A structured literature review search was conducted to identify and synthesise evidence on the impact of learning technologies on the learning environment of medical students in Africa (Appendices). Electronic database searches were performed using Ovid Medline, Embase, and Emcare following a systematic search strategy, as shown in Figure 1. The search combined three main concepts: (1) population - medical students; (2) setting - African countries; and (3) intervention - learning technologies related to medical education.

The following search strategy was applied: (“medical students” OR “undergraduate medical students” OR “medical undergraduates”).ti,ab,sh. Combined with African context terms (e.g., South Africa, Africa, Northern Africa, Central Africa, Eastern Africa, Western Africa, Southern Africa).ti,ab,sh. Technology-related terms (e.g., technology, virtual reality, artificial intelligence, AI, social media, e-learning, digital learning platforms, virtual classroom, simulation).ti,ab,sh. Educational terms (e.g., learning, teaching, education).ti,ab,sh.

The Boolean logic applied was (“medical students” OR “undergraduate medical students” OR “medical undergraduates”) AND (Africa OR regional African terms) AND (technology OR virtual reality OR artificial intelligence OR AI OR social media OR e-learning OR simulation OR digital learning platforms OR virtual classroom) AND (learning OR teaching OR education).

This strategy yielded 363 citations. After removing duplicates, titles and abstracts were screened independently by two reviewers. Full texts were retrieved for studies that met the inclusion criteria or where eligibility was uncertain. Reference lists of included articles were hand-searched to identify additional relevant studies.

Inclusion and Exclusion Criteria

Studies were eligible if they examined learning technologies used by medical students in African countries. Only English-language studies were included. Studies focusing exclusively on non-medical disciplines, postgraduate training, opinion pieces, and conference abstracts were excluded.

Study Selection and Data Extraction

Two reviewers independently screened titles, abstracts, and full texts. Disagreements were resolved by consensus or consultation with a third reviewer. Data extracted included study design, setting, type of technology used, learning environment features, outcomes measured, and key findings, as well as limitations/challenges.

Quality Assessment and Data Synthesis

Methodological quality was assessed using appropriate critical appraisal tools: the Joanna Briggs Institute (JBI) Critical Appraisal Checklists for observational and qualitative studies. Due to heterogeneity in study designs and outcome measures, a narrative synthesis of findings was performed, with descriptive statistics reported where appropriate.

Results

The initial search yielded 363 papers. Once duplicates were removed and papers excluded. A total of 14 papers were included in the study.

Geographic Distribution of Studies

The included studies in the review were conducted across seven African countries, as shown in Table 1. The majority of studies originated from South Africa (n = 4), followed by Nigeria (n = 3) and Egypt (n = 3). Other regions represented were Uganda (n = 1), Kenya (n = 1), Zambia (n = 1), and Somaliland (n = 1). One study conducted its initial pilot study in Nigeria, then expanded the initiative to other LMIC in Africa and Asia through online outreach. Notably, three studies reported initiatives implemented in collaboration with international institutions such as the United States and the United Kingdom. Most studies employed cross-sectional or mixed methods study designs. 

**Table 1:** Distribution of studies by geographic location

Articles	Country	Number of Studies (n)
Enoch et al. [21], Wever et al. [29], Lazarus et al. [31], Ebrahim et al. [32]	South Africa	4
Posever et al. [22], Onyeka et al. [28], Anyanwu et al. [33]	Nigeria	3
Amgad et al. [23], Al-Neklawy et al. [24], Negm et al. [26]	Egypt	3
Fant et al. [25]	Kenya	1
Ayoola et al. [27]	Uganda	1
Murphy et al. [30]	Somaliland	1
Ezeala et al. [34]	Zambia	1

The study populations consisted of undergraduate medical students, spanning from preclinical years to final year, with a single study including paramedic students. Around 20% of the studies involved graduates (residents and house officers), and one focused on clinical teaching staff engaged in curriculum development and simulation delivery.

Learning Technology

A wide variety of digital learning technologies were employed across the included studies, as shown in Table 2. The most frequently used were live conferencing platforms such as Zoom (Zoom Video Communications, San Jose, CA), WizIQ (WizIQ Pvt. Ltd., Bangalore, India), and YouTube (Google LLC, Mountain View, CA), reported in six studies [21-26]. These supported synchronous delivery of lectures, case-based discussions, and clinical skills demonstrations, often with recordings made available for asynchronous review.

**Table 2:** Type of learning technology utilised

Citation	Type of Learning Technology
Enoch et al. [21]	Zoom Learn 2021 (asynchronous lectures)
Posever et al. [22]	Synchronous live tutorials on Zoom; GMEC website; Google Drive; Email; Social media
Amgad et al. [23]	Multiple Web 2.0 tools (Google Drive, Dropbox, Mendeley, Facebook, Google Hangout, YouTube, REDCap, Moodle)
Al-Neklawy et al. [24]	WizIQ virtual classroom (live synchronous lectures with recordings for asynchronous viewing)
Fant et al. [25]	Online collaborative platform (specific platform not stated)
Negm et al. [26]	Pre-recorded asynchronous lectures distributed through email and Zoom
Ayoola et al. [27]	App-based e-learning with online videos (Digital Medic App)
Onyeka et al. [28]	VTR mobile app (accessible via mobile phones and desktop computers)
Wever et al. [29]	YouTube educational videos (Ucteach Ortho channel)
Murphy et al. [30]	Online peer-to-peer e-learning platform using MedicineAfrica (instant messaging for real-time communication)
Lazarus et al. [31]	Tablets preloaded with anatomy content
Ebrahim et al. [32]	Zoom (live tutorials, CBDs); Moodle LMS; asynchronous tools (PowerPoint, quizzes, recorded skill videos)
Anyanwu et al. [33]	SLTDVM (Tagged Digital Virtual Microscopy): 250+ virtual histology slides compiled in PowerPoint with varied images and stains
Ezeala et al. [34]	Computer-based simulation platform (CyberPatient, OBSim, AutonomiCAL, Virtual Cat, RatCVS)

Several studies examined mobile applications, notably the Digital Medic app (Stanford Center for Health Education, Stanford University, Stanford, CA) and VTR Mobile (InStrat Global Health Solutions, Abuja, Nigeria), to provide asynchronous access at home [27-28]. Web 2.0 tools and learning management systems (LMS) (e.g., Google Drive (Google LLC, Mountain View, CA), Dropbox (Dropbox Inc., San Francisco, CA), GMEC website (Global Medical Education Collaborative, Boston, MA), Learn2021 (University of KwaZulu-Natal, Durban, South Africa)) were used to facilitate content delivery, collaboration, and resource sharing [21,22,23,25,29,30]. Three studies specifically leveraged online collaborative platforms to connect African and LMIC students with international participants, enabling real-time communication, PBL, mentorship, and demonstrative videos [22,25,30].

Other innovative technologies included tablet-based anatomy resources [31], computer-based pharmacology simulations [32], and virtual histology slides [33]. Many of these initiatives were introduced during the COVID-19 pandemic to mitigate disruption to face-to-face teaching.

Educational Outcomes

Digital learning technologies consistently demonstrated positive impacts on knowledge acquisition, clinical skills, and learner confidence. Enoch et al. [21] reported that blended learners outperformed both online-only and pre-pandemic traditional cohorts across psychomotor, affective, and cognitive domains, achieving median scores of 90% (95% CI 86-92), 82% (95% CI 80-85), and 87% (95% CI 84-90), respectively. Onyeka et al. [28] also found significant improvements in learner performance, with median test scores rising from 40 to 60 after module completion (p < 0.001). Similarly, Anyanwu et al. [33] demonstrated better performance using virtual histology slides compared with traditional microscopy (52.3 ± 18.2 vs 48.7 ± 16.8, p < 0.001).

Multiple studies concluded that blended approaches were preferred and superior to either fully online or traditional teaching, bridging gaps between theory and practice while enhancing engagement [21,23,24]. Online collaborative initiatives [22,25,30] further showed potential for scalability, connecting large numbers of students and tutors across LMICs and high-income countries at low cost through existing infrastructure and volunteer networks.

Assessment Methods

Assessment strategies varied across each study but fell broadly into two categories: objective performance measures and self-reported qualitative outcomes. Objective assessments included OSCEs, pre-/post-test quizzes [28,32], module completion tests [28], laboratory reports and assignments [23,34], and practical examinations [33]. In contrast, qualitative measures such as surveys, interviews, and reflections [23,27,31] focused on learner confidence, engagement, satisfaction, and challenges.

Challenges and Barriers

Although digital learning technologies were well received and effective in improving outcomes, they did not come without their challenges that can be attributed to limited resources, infrastructure, and digital literacy in the African countries. Poor internet connectivity and limited device access frequently disrupted participation in both synchronous and asynchronous learning, leading to reduced satisfaction [22,24,28,30,31]. Furthermore, high mobile data costs also hindered sustained engagement [22,28]. Inconsistent computer access was reported in international collaborative initiatives [30]. Some challenges specific to online collaborative efforts included language barriers, difficulty scheduling meetings across time zones and the absence of real-time video exchange, hindering cultural exchange [30].

Poor digital literacy also emerged as a significant barrier to learning technology initiatives uptake, with some students unfamiliar with new platforms [22,31]. In fact, Posever et al. [22] reported that 8% of learners had a limited understanding and comfort level of platform use. Technical issues with specific tools were also highlighted, including app access difficulties [28], unstable video exchange [30], and platform glitches [23,24]. Additional COVID-related challenges included personal stressors [32], reduced student participation [21], and frustration with remote learning [22]. A summary of the learning technology, key outcomes and challenges is shown in Table 3.

**Table 3:** The region, population, medical domain, type of learning technology, as well as the key outcomes and challenges of each study

Citation	Region	Population and Domain	Learning Technology	Key Outcomes	Limitations / Challenges
Enoch et al. [21]	South Africa	Third-year students - Clinical Skills	Zoom (asynchronous lectures)	• Blended learners outperformed online and pre-pandemic cohorts	• Low participation • Examiner shortages • Selection bias
Posever et al. [22]	Nigeria / LMIC	Pre-clinical and clinical students - Clinical Skills	Zoom tutorials, website, email, social media	• 91% improved confidence • Strong tutor ratings • Identified skill gaps	• Poor internet • Power outages • Self-selection bias
Amgad et al. [23]	Egypt	Undergraduates and graduates - Research, Bioinformatics	Web 2.0 tools (Google Drive, Moodle, YouTube, etc.)	• High engagement (82.6%) • 82.7% found course appropriate	• Technical issues • Long surveys • Self-report bias
Al-Neklawy et al. [24]	Egypt	First-year students - Embryology	WizIQ virtual classroom	• 90% recommended course • 81% found online learning comparable/superior	• Technical access issues • Limited assessments • Internet dependency
Fant et al. [25]	Kenya/USA	Faculty and students – Paediatrics	Online collaborative simulation	• Improved debriefing and confidence • Positive feedback	• Small sample • Time constraints
Negm et al. [26]	Egypt	Final-year students and house officers - Radiology	Pre-recorded lectures, Zoom	• >97% improved understanding • Significant pre/post-test gains	• Missing demographic data • Limited graduate participation
Ayoola et al. [27]	Uganda	Fifth-year students and Emergency Medicine (EM) residents - Emergency	Digital Medic App (e-learning)	• Improved foundational knowledge • Preference for blended model	• Selection bias • Limited contextualisation • Lack of local mentorship
Onyeka et al. [28]	Nigeria	Fifth-year students - Pain Management	VTR mobile app	• 33% module completion • Improved post-test scores	• Poor integration • Connectivity issues • Selection bias
Wever et al. [29]	South Africa	Fifth-year students - Orthopaedics	YouTube videos	• 337,000 views • Procedural videos most popular	• Limited viewer data • No outcome measures • Reliance on analytics
Murphy et al. [30]	Somaliland/UK	Final-year students - Psychiatry	MedicineAfrica platform	• Improved mental health understanding • Cultural exchange benefits	• Small sample • Technical issues • Scheduling barriers
Lazarus et al. [31]	South Africa	Second-year students - Anatomy	Tablets with anatomy content	• Flexible, self-paced learning • Greater access for rural students	• Poor Wi-Fi • Unfamiliarity with tablets • Limited generalisability
Ebrahim et al. [32]	South Africa	Sixth-year students - Surgery	Zoom, Moodle, interactive videos	• High engagement • Deep learning linked with motivation	• Pandemic stress • Low discussion engagement • Convenience sampling
Anyanwu et al. [33]	Nigeria	Undergraduates - Histology	Virtual microscopy (SLTDVM)	• Higher scores vs traditional microscopy • Widely supported	• Cannot replace practical skills • Limited virtual tools
Ezeala et al. [34]	Zambia	Undergraduates - Pharmacology	Computer-based simulations	• Improved understanding • Promoted critical thinking	• Limited hands-on learning • Tech dependency • Oversimplified models

Learner Perceptions

Learner feedback was overwhelmingly positive, although four studies did not formally report perceptions [21,29,32,34]. Where data were available, students consistently reported high satisfaction [21,23,24,26] and engagement [22-24,26,28,29,31,32]. Learners valued flexibility, accessibility, and self-paced study [24,28,31], with additional benefits noted for specific groups such as visually impaired students [33]. Confidence and interest in subject matter often increased, and students expressed enthusiasm for mentorship and cross-cultural collaboration in international initiatives [22,25,30].

Nonetheless, negative experiences were also documented, including frustration with technical issues, dissatisfaction with limited practical lab skills training [27,33], and preferences for traditional methods in anatomy teaching, such as dissections [31].

Discussion

Effectiveness of Learning Technology Use in Medical Education

The use of digital learning technologies to mitigate limited institutional resources in LMICs has been trialled and shown to be effective across multiple cohorts, according to a review conducted by Frehywot et al. [35]. In our review of learning technologies used in medical education in African countries, the most common modalities were video-conferencing platforms, such as Zoom and WizIQ, for synchronous teaching, LMS that are specialised software platform designed to deliver, manage, and track educational content [36], tools, such as Learn2021, Google Drive, and the GMEC website, were used to host interactive content and assessments; and mobile applications (e.g., Digital Medic, VTR) for asynchronous, self-paced study. Across these interventions, we found consistent reports of knowledge gain and high learner satisfaction, spanning diverse competencies including Pap smear technique, OSCE and history taking, histology, and radiology interpretation [21,22,26,33].

Our findings align with international evidence that underscores the flexibility and utility of LMS platforms as key drivers of successful outcomes. For example, Chen et al. [36] demonstrated that 94.22% of students reported satisfaction with LMS during the COVID-19 pandemic, with perceived usefulness and ease of use as the strongest predictors of satisfaction. Similarly, an LMIC study evaluating LMS reported high satisfaction scores (4.53/5) and completion rates (90%), attributing success to both usability and structural incentives such as integration with credit and attendance marks [37]. Together, these findings suggest that in African LMIC contexts, LMS design should prioritise mobile-friendliness, optimisation for low-bandwidth environments, and integration with assessment and accreditation systems to translate satisfaction into sustained engagement and completion.

These results can be further understood through the lens of the technology acceptance model (TAM), which posits that two key factors-perceived usefulness and perceived ease of use-determine users’ acceptance and continued utilisation of technology [38]. In several included studies, learner satisfaction and engagement were closely associated with these dimensions, as students reported higher motivation when platforms were intuitive, accessible, and perceived as directly relevant to their learning needs [21,23,27,32]. This suggests that technology adoption in African medical education is not solely a function of infrastructure but also of users’ attitudes and behavioural intention to use technology. Incorporating TAM-informed design principles could therefore enhance both uptake and sustainability of digital learning initiatives in resource-limited contexts.

Mobile applications also showed strong potential as complements to medical education. A systematic review of 52 studies confirmed that mobile apps enhance knowledge and skills, with features such as offline access and low cost cited as practical enablers of uptake and persistence [39]. These advantages were mirrored in our dataset, where learners valued asynchronous access for its convenience and ability to support self-paced study [22,27]. Similar benefits have been reported internationally, with mobile apps successfully supporting surgical training [40], cardiopulmonary resuscitation [41], and clinical decision-making [42], all showing significant improvements in learning outcomes.

Use of Blended Learning Approaches

Despite these strengths, limitations were evident when technology was used as a replacement rather than a supplement to traditional teaching. Learners consistently valued blended approaches, where digital platforms provided a strong foundation of knowledge while in-person teaching offered essential hands-on experience. For instance, Ayoola et al. [27] found that learners preferred blended approaches for emergency medicine training, and Enoch et al. [21] demonstrated objectively superior outcomes when blended methods were employed. Simulation programmes [34] and virtual microscopy [33] were praised for accessibility but criticised for inadequately supporting practical skill development and for their inability to replace wet-lab or hands-on training. Students in these studies consistently favoured hybrid approaches combining virtual and traditional resources.

This preference aligns closely with the Community of Inquiry (CoI) framework, which conceptualises effective online and blended learning as the intersection of cognitive presence, social presence, and teaching presence [43]. Within our reviewed studies, successful learning environments reflected these three elements: structured instructional guidance (teaching presence) through live tutorials and moderated forums; peer collaboration and interaction (social presence) via group discussions and virtual teamwork; and reflective, self-directed engagement with content (cognitive presence) through asynchronous modules and self-assessment. The CoI model thus provides a useful theoretical lens for interpreting why blended approaches appear particularly effective in African medical education, combining the social support of traditional classrooms with the flexibility and autonomy afforded by digital tools.

This is consistent with Wilcha et al.’s [44] review of virtual education during the COVID-19 pandemic, which cautioned that online teaching alone often provides weak clinical skill foundations, particularly in history taking and physical examinations [45], ultimately resulting in poor preparation for clinical practice.

Contextual and Institutional Factors

The effectiveness of learning technology in LMIC medical education is also contingent on institutional and infrastructural readiness. Frehywot et al.’s [35] review of e-learning in LMICs, largely drawn from Brazil, India, and South Africa, highlighted common barriers including unreliable connectivity, limited device access, high data costs, and variable digital literacy. These challenges were similarly reported in our included African cohorts. It is therefore essential to address such structural barriers and critically assess institutional capacity before implementing educational interventions.

Furthermore, local curricular alignment and clinical relevance were shown to be equally important [27], emphasising the need to adapt technologies to local context and clinical realities rather than directly transplanting models from high-income settings. 

Limitations of This Review

Our review provides a focused synthesis of learning technology in African medical education and evaluates a diverse range of educational interventions, which strengthens the applicability of its findings. However, several limitations may affect the validity of our conclusions. Many studies relied on self-reported outcomes through surveys and interviews, with few employing objective outcome measures such as validated assessment scores or blinded evaluations. This leaves room for response bias and potential overestimation of intervention impact.

Most studies were also limited by single-centre designs and voluntary participation, meaning students with a greater interest in the subject area may have been more likely to participate, potentially inflating levels of satisfaction and engagement. Moreover, the absence of comparative control groups in many interventions limits the ability to establish causal inferences between learning technologies and observed outcomes. Few studies directly addressed the impact of online-only modalities on practical skill acquisition, which traditionally relies on hands-on, in-person training.

Finally, none of the included studies reported long-term follow-up, leaving gaps in understanding knowledge retention, sustained skill development, and eventual impact on clinical practice.

Future Directions

Our review highlights important gaps that future research should address. Multi-centre trials conducted across diverse African contexts are needed to enhance generalisability beyond single-institution studies. Furthermore, standardised outcome measures - including knowledge acquisition, practical skill competence, learner confidence, and cost-effectiveness - would improve comparability across interventions.

As our review shows, blended learning approaches positively influence the learning environment of African medical students, but infrastructural barriers such as poor internet connectivity remain major challenges. Future research should evaluate offline-compatible and low-bandwidth solutions to ensure equitable access.

While most studies included in this review focused on Web 2.0 technologies - such as video conferencing, LMS platforms, and mobile applications that prioritise interactivity and accessibility - the next wave of innovation lies in Web 3.0 tools. Artificial intelligence-driven adaptive platforms, virtual reality (VR), and augmented reality (AR) remain underexplored in low-resource settings and warrant rigorous evaluation to determine their feasibility, scalability, and educational value in African medical education.

## Conclusions

Learning technologies hold enormous potential to transform medical education in Africa, enhancing access, equity, and quality of training, whilst also improving the learning environment. Yet this potential remains constrained by systemic barriers, from infrastructure limitations to pedagogical gaps. While isolated successes demonstrate feasibility, the broader challenge lies in achieving sustainable, scalable integration. With targeted investment, capacity building, and locally driven innovation, African medical schools can harness digital tools not only to overcome historical limitations but also to leapfrog into more equitable and globally connected models of medical education.

## Appendices

**Table 4:** PRISMA 2020 checklist PRISMA: Preferred Reporting Items for Systematic Reviews and Meta-Analyses

Section/Topic	Item No.	PRISMA 2020 Checklist Item	Reported in Manuscript
Title	1	Identify the report as a systematic review.	Title page
Abstract	2	Provide a structured summary including background, objectives, data sources, eligibility, appraisal, results, and conclusions.	Abstract
Introduction	3	Describe the rationale for the review in the context of existing knowledge.	Introduction
Introduction	4	Provide an explicit statement of the objectives or questions the review addresses.	Introduction, final paragraph
Methods	5	Specify inclusion and exclusion criteria and how studies were grouped for synthesis.	Inclusion and Exclusion Criteria section
Methods	6	Specify all information sources (databases, registers, websites, etc.) used to identify studies.	Search Strategy section
Methods	7	Present the full search strategies for all databases, including limits and date ranges.	Search Strategy section
Methods	8	Specify the process for selecting studies (screening, eligibility, inclusion).	Study Selection and Data Extraction section
Methods	9	Describe the data collection process, including how data were extracted and verified.	Study Selection and Data Extraction section
Methods	10	List and define all outcomes sought (primary and secondary).	Quality Assessment and Data Synthesis section
Methods	11	Specify methods used to assess risk of bias in included studies.	Quality Assessment section
Methods	12	Describe methods of analysis and synthesis.	Quality Assessment and Data Synthesis section
Results	13	Provide the number of studies screened, assessed, and included, with reasons for exclusions.	Figure 1 (PRISMA diagram)
Results	14	Present characteristics of included studies.	Results, Tables 1-3
Results	15	Present results of individual studies, including summary data for each outcome.	Results section
Results	16	Present results of risk of bias assessments.	Results
Results	17	Present results of all syntheses (thematic or quantitative).	Results and Discussion sections
Discussion	18	Summarise main findings, including strength of evidence and relevance to key groups.	Discussion, first two subsections
Discussion	19	Discuss limitations of the evidence included in the review.	Limitations of This Review subsection
Discussion	20	Discuss limitations of the review process itself.	Limitations subsection
Discussion	21	Provide a general interpretation of results in context of other evidence.	Discussion section
Other information	22	Describe sources of funding and the role of funders.	None
Other information	23	Report any competing interests.	Disclosures (none)
Other information	24	Provide registration information for the review (if registered).	Not registered
